# The Performances of Hyperspectral Sensors for Proximal Sensing of Nitrogen Levels in Wheat

**DOI:** 10.3390/s20164550

**Published:** 2020-08-13

**Authors:** Huajian Liu, Brooke Bruning, Trevor Garnett, Bettina Berger

**Affiliations:** The Plant Accelerator, Australian Plant Phenomics Facility, School of Agriculture, Food and Wine, University of Adelaide, Waite Campus, Building WT 40, Hartley Grove, Urrbrae SA 5064, Australia; brooke.bruning@adelaide.edu.au (B.B.); trevor.garnett@grdc.com.au (T.G.); bettina.berger@adelaide.edu.au (B.B.)

**Keywords:** wheat, nitrogen, hyperspectral imaging, plant phenotyping, partial least square regression

## Abstract

The accurate and high throughput quantification of nitrogen (N) content in wheat using non-destructive methods is an important step towards identifying wheat lines with high nitrogen use efficiency and informing agronomic management practices. Among various plant phenotyping methods, hyperspectral sensing has shown promise in providing accurate measurements in a fast and non-destructive manner. Past applications have utilised non-imaging instruments, such as spectrometers, while more recent approaches have expanded to hyperspectral cameras operating in different wavelength ranges and at various spectral resolutions. However, despite the success of previous hyperspectral applications, some important research questions regarding hyperspectral sensors with different wavelength centres and bandwidths remain unanswered, limiting wide application of this technology. This study evaluated the capability of hyperspectral imaging and non-imaging sensors to estimate N content in wheat leaves by comparing three hyperspectral cameras and a non-imaging spectrometer. This study answered the following questions: (1) How do hyperspectral sensors with different system setups perform when conducting proximal sensing of N in wheat leaves and what aspects have to be considered for optimal results? (2) What types of photonic detectors are most sensitive to N in wheat leaves? (3) How do the spectral resolutions of different instruments affect N measurement in wheat leaves? (4) What are the key-wavelengths with the highest correlation to N in wheat? Our study demonstrated that hyperspectral imaging systems with satisfactory system setups can be used to conduct proximal sensing of N content in wheat with sufficient accuracy. The proposed approach could reduce the need for chemical analysis of leaf tissue and lead to high-throughput estimation of N in wheat. The methodologies here could also be validated on other plants with different characteristics. The results can provide a reference for users wishing to measure N content at either plant- or leaf-scales using hyperspectral sensors.

## 1. Introduction

Wheat (*Triticum aestivum*) accounts for the majority of Australia’s grain production and is one of the most important grain crops worldwide. Australia produces around 22 million tonnes annually with a gross value over AU$ 6 billion [1]. The sustainable improvement of wheat yield is a major research focus to meet the increasing demand of wheat over the next 20 years. Nitrogen is a critical driver for crop yield and healthy plants generally contain 3–4% N in their above-ground tissue [2]. Nitrogen is a major component of chlorophyll, the compound by which plants use sunlight energy to produce sugars from water and carbon dioxide. i.e., photosynthesis. It is also a major component of amino acids, the building blocks of proteins. Nitrogen is a component of energy-transfer compounds, such as adenosine triphosphate, allowing cells to conserve and use the energy released in metabolism. Finally, nitrogen is a significant component of nucleic acids such as DNA, the genetic material that allows cells to grow and reproduce [3]. Effective diagnosis and management of plant N status are vital to maximising yields and are best based on real-time monitoring of biomass and plant nitrogen levels. Traditionally, N measurement requires destructive methods whereby plant samples are first dried and ground before being analysed using combustion-based methods. This laboratory approach is both time-consuming and expensive, particularly for a large number of samples [4].

Recently, hyperspectral sensors have demonstrated robust and accurate quantification of N content in plants [5]. Hyperspectral sensors operating in the visible and near-infrared (VNIR) wavelength range of 450 nm to 10,000 nm contain either charge-coupled devices (CCDs) or complementary metal-oxide-semiconductor (CMOS) photonic detectors. Those sensitive to the short-wave infrared wavelengths (SWIR) of 1000 nm to 2500 nm use photonic detectors composed of indium gallium arsenide (InGaAs) or mercury cadmium telluride (MCT) [6]. Early studies were conducted using non-imaging optical spectrometers, often simply called spectrometers, recording the intensity, reflectance or transmittance of light as a function of wavelength or frequency at a single point [7]. Spectrometers can be flexibly configured to work in either the full range or sub-regions of the 400 nm to 2500 nm spectrum with high spectral resolution. For each measurement, the output is a vector, usually called a spectral signature, recording the average value of intensity, reflectance or transmittance in the field of view (FOV) of the lens. Different spectrometers have been investigated to measure leaf N content in wheat [8,9,10], maize [11] and tobacco [12]. The fast-developing area of hyperspectral imaging integrates imaging and spectroscopy technologies and provides both visual representations of targets as well as the corresponding spectral information. The integration of spatial and spectral information has enabled the merging of computer vision technologies and spectroscopy, providing enriched datasets for the non-destructive measurement of plants. In contrast to spectrometers, hyperspectral cameras usually have limited spectral ranges and wider bandwidths. Hyperspectral cameras operating in the SWIR spectrum usually have coarser spectral and spatial resolutions than VNIR cameras and are more expensive, heavier and have a shorter lifetime than VNIR cameras. In proximal sensing, hyperspectral cameras have been used to estimate N in wheat [4,13] and pepper [14]. In aerial remote sensing, hyperspectral systems have been successfully used to measure the N content of wheat [15], mangrove [16] and maize [17].

Although previous studies have demonstrated the feasibility of using hyperspectral techniques for estimating plant N level, the sensors and configurations differed in different studies, making the transfer of the methods difficult. To our knowledge, there has been no report comparing the performances of different hyperspectral sensors operating in different wavelength ranges, band centres and bandwidths. This study aims to evaluate the performances of different hyperspectral sensors for N quantification in wheat. Our study demonstrated that proximal sensing of N in plants using hyperspectral sensors is heavily affected by system setups. Hyperspectral imaging systems with ideal system setups could outperform non-imaging spectrometer with contact measurement. Our study confirmed that high-throughput and non-destructive estimation of N in wheat is possible by adopting hyperspectral imaging technologies. We evaluated the responses of different photonic detectors and spectral resolutions and the results can guide users to select correct sensors for their applications. The model-selected wavelengths most correlated to N, i.e., key-wavelengths, have physical meanings; thus the models are explainable. Besides that, only using the key-wavelengths can significantly reduce computation without losing accuracy. The methodologies proposed here could also be generalised to other crops and the results can provide a reference for users wishing to measure N content at either plant- or leaf-scale using hyperspectral sensors.

Section 2 describes the methods for evaluating the sensors. Section 3.1, Section 3.2, Section 3.3 and Section 3.4 answer the four research questions respectively: (1) How do hyperspectral sensors with different system setups perform when conducting proximal sensing of N in wheat leaves and what aspects have to be considered for optimal results? (2) What types of photonic detectors are most sensitive to N in wheat leaves? (3) How do the spectral resolutions of different instruments affect N measurement in wheat leaves? (4) What are the key-wavelengths with the highest correlation to N in wheat? Section 4 concludes this study.

## 2. Materials and Methods

### 2.1. Wheat Plants

The five most widely-grown wheat varieties, Gladius, Axe, Scepter, Corack and Yitpi (Table A1) were selected for the study. The leaves of these wheat varieties exhibit different morphological and spectral features at the growing stage of 40 to 60 days. The plants were grown in soil substrate under four N treatments of 25, 50, 100 and 200 mg N/kg with ten biological replicates, resulting in a total of 200 pots of individual wheat plants. The soil was a 1:1:1 (v:v:v) mixture of UC Davis (University of California, Davis) -mix: coco-peat: clay-loam without N fertiliser. The pH was balanced to 6.4 using dolomite lime. The pots were 150 mm in diameter and each pot contained 2.7 kg soil. On 3 September 2019 (day 0), the seeds were sown and then the pots were kept in a greenhouse with regular watering. On 24 September 2019 (day 21), the pots were transferred to the conveyor system of an automated plant phenotyping platform (LemnaTec GmbH, Aachen, Germany) at The Plant Accelerator, University of Adelaide. The pots occupied 10 lanes × 20 positions which were split into two sides. Each side was split into five lane-pairs, each comprising 2 lanes × 10 positions. Each lane-pair comprised five main units of 2 × 2 = 4 ports each. Within each side, the five varieties were allocated to the 5 × 5 main units using a Latin square. The four nitrogen treatments were randomised to the four pots within each main unit. The whole design was randomised using the R package dae [18]. The N treatments were applied to the plants on the day of loading (day 21) using liquid ammonium nitrate (AN). Each pot was manually watered 150 mL liquid AN with the concentration of 1.17, 2.34, 4.68 and 9.36 mg/mL for the four N treatments respectively. Once on the conveyor system, the plants were automatically watered daily to a set target weight.

### 2.2. Data Collection

**Figure A1:**
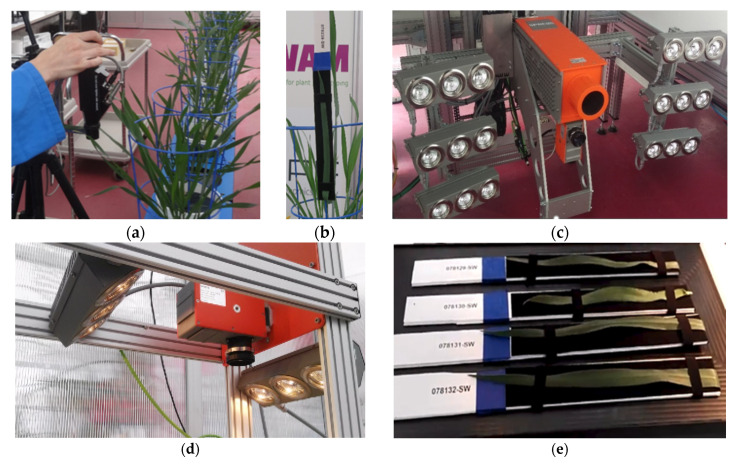
Data collection. (**a**) Data collection using the ASD Spec 3 spectrometer. (**b**) A wheat leaf bound to a leaf-bed for imaging. (**c**) The system set up of the hyperspectral imaging system in the phenotyping platform. The larger camera on the top is the SWIR camera and the smaller camera on the bottom is the FX10 camera. (**d**) The system set up of the labscanner using the FX10 camera. (**e**) The leaves bound to the leaf-beds and laid on the conveying tray of the labscanner for imaging.

The hyperspectral data were collected using a non-imaging spectrometer, two hyperspectral cameras in a plant phenotyping platform and a hyperspectral camera in a benchtop labscanner. The data were collected at three time-points on 17 and 18 October 2019 (day 44 and 45), 24 and 25 October 2019 (day 51 and 52) and 31 October and 1 November 2019 (day 58 and 59). The spectrometer (ASD FieldSpec 3, Analytic Spectral Devices, Boulder, USA) was equipped with a leaf clip holder (Figure A1a). A mask was attached to the end of the probe to reduce its FOV and allow measurement of narrow leaves. The ASD FieldSpec 3 integrates three sensors and can measure the spectra from 350 to 2500 nm. The full width at half maximum (FWHM) is 3 nm from 350 nm to 1000 nm and 10 nm from 1000 nm to 2500 nm. The output reflectance values were resampled to 1 nm FWHM. A 100W halogen light enabled constant illumination inside the leaf clip holder. For each plant, a spectrum was recorded in the central region of a fully expanded young leaf.

To track the leaves measured by the spectrometer, the measured leaf was marked immediately after each spectrometer measurement and then bound to a leaf bed (Figure A1b). The front of the leaf bed is composed of the black background, blue marker and sample ID. The black material minimised the background reflection while the blue maker was used to detect the leaf bed in the collected hyperspectral images. The leaf beds were positioned to directly face the cameras, to reduce the effects of varying leaf angles.

Two cameras were installed in the hyperspectral imaging chamber (WIWAM, Ghent, Netherlands) of the phenotyping platform for whole-plant imaging (Figure A1c). The FX10 camera (Specim, Oulu, Finland) captured the VNIR data from 400 nm to 1000 nm with 5.5 nm FWHM while the SWIR camera (Specim, Oulu, Finland) operated in the range of 1000 nm to 2600 nm with 12 nm FWHM. The hyperspectral imaging chamber was illuminated by two 3 × 3 halogen light arrays to ensure consistent illumination across the full wavelength range. The working distance from the cameras to the plants was approximately 1.4 m. The FX10 camera had a 38° lens with 512 pixels per line and the SWIR camera had a 34° lens with 384 pixels per line, resulting in 2.6 mm and 2.1 mm horizontal spatial resolution respectively.

In addition to the FX10 camera used in the plant phenotyping platform, another one was mounted on a benchtop labscanner for imaging individual leaves (Figure A1d). The illumination was provided by two 3 × 1 halogen light arrays on the sides of the camera. The camera was equipped with a 38° lens and the working distance from the lens to the conveying tray was 160 mm, resulting in 0.13 mm horizontal spatial resolution. Immediately after the hyperspectral imaging in the phenotyping platform, the leaves bound to the leaf-beds were detached from the plants and then laid on the conveying tray for scanning (Figure A1e). After the hyperspectral data collection, the leaves were dried in a 60 °C oven for 48 h and then sent to a laboratory (Australian Precision Ag Laboratory, Adelaide, Australia) for reference N% analysis [4].

### 2.3. Data Processing

**Figure 1:**
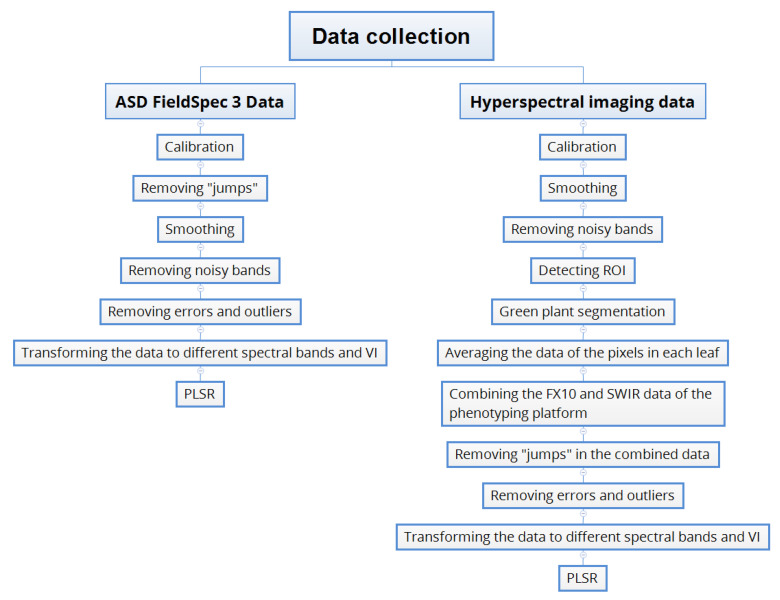
Workflow of hyperspectral data processing.

The data collected by the individual cameras and spectrometer were processed separately in Python 3.6 [19]. The reflectance data were pre-processed and transformed into different spectral bands with different band centres and bandwidths. Then the transformed data were used to train and validate partial least square regression (PLSR) models for estimating N content. The workflow is shown in Figure 1 and detailed below.

#### 2.3.1. Pre-Processing

##### Pre-Processing of the FieldSpec 3 Data

The FieldSpec 3 data were calibrated using the software provided by the manufacture. Because spectral data were collected from three different photonic detectors inside the FieldSpec3, the adjacent values across the different sensors were not continuous, causing “jumps” in the data which needed to be removed. Suppose the data from the first sensor includes *n* spectral bands and the *m* neighbouring values from n-m to n are linear. A straight line can be fitted using least square regression so that the *n*+1 value of the first sensor can be estimated. This estimated value is considered to be the first value of the second sensor and the remaining values are aligned to this value. All of the jumps in a spectral signature can be removed this way. In this experiment, m was set to six to obtain the best results. The random noise of the spectral signatures from the sensor was smoothed using the Savitzky–Golay filter [20]. After smoothing, the values below 400 nm and above 2400 nm were still noisy, and were therefore removed.

##### Pre-Processing of the Hyperspectral Images

The imaging data were calibrated using Equation (1) [6,21]

(1) $$r_{p}\left(\lambda ,\;x,\;y\right)=\frac{i_{p}\left(\lambda ,\;x,\;y\right)-i_{d}\left(\lambda ,\;x,\;y\right)}{i_{w}\left(\lambda ,\;x,\;y\right)-i_{d}\left(\lambda ,\;x,\;y\right)}$$

where *r*_p_ is the reflectance values of the plant at the spatial location (*x*, *y*) and the wavelength λ. *i*_p_, *i*_d_ and *i*_w_ represent the measured image intensity values of the plant, dark current and white reference respectively. After calibration, the same method described above was used to smooth and remove noisy bands from the hyperspectral images. In the images, the pixels of the leaves bound to the leaf-beds needed to be extracted. First, random pixels of the blue markers were selected and their spectral signatures were averaged to form a target signature. For each image, the spectral angles between each pixel and the target signature were calculated. The pixels with spectral angles smaller than a threshold of 0.2 were classified as blue marker. Through the location and angle of the blue marker, the ROI of the leaf-bed was then automatically detected. In the ROI, the pixels of the leaf were separated from the background using a green plant segmentation algorithm proposed by Liu, et al. [21]. The spectral signatures were first transformed to hyper-hue [22,23] and then a one-class support vector machine was trained to do the classification. After segmentation, the spectral signatures of the pixels in each of the bounded leaves were averaged. The FX10 and SWIR data of the phenotyping platform was combined to form a full range spectral signature and the “jumps” between the two sensors were removed.

##### Outlier Removal and Data Cleaning

The 200 plants were measured at three time-points, resulting in 600 samples of reflectance data and the corresponding reference N% for each hyperspectral sensor. However, some data had to be removed due to outliers of N% values or data acquisition errors (Table A2). A total of 42 sampled leaves were too small (dried leaf weight < 100 mg) to return laboratory analysis of N%, and therefore were removed. There were eight samples whose N% values were either below 1% or above 7% and were considered as outliers. In addition, seven data samples had to be excluded due to errors in image acquisition or segmentation. This resulted in a total of 543 valid samples for further analysis. The pre-processed data and python codes for demonstration is public available (refer to Supplementary Materials).

#### 2.3.2. Partial Least Square Regression

Multivariate regression is a method used to measure the degree of linear relationship between multiple independent variables (predictors) and dependent variables (responses). While many different multivariate regression algorithms exist, PLSR has proven to be most robust for N measurement using high-dimensional hyperspectral data [8,9,10,11,12,13,14]. In a preliminary analysis, we tested several well-accepted multivariate regression algorithms which have previously been used for N measurement in plants, including support vector machine regression [16] and stepwise regression [15], and found that PLSR achieved equal or better results. Therefore, the PLSR algorithm was selected for regression. Five-fold cross-validation was conducted to train and validate the models (refer to Figure A4 for the distributions of the training and validation data). The number of latent variables was automatically selected in the range of 1 to 20 to get the lowest regression errors.

**Figure 2:**
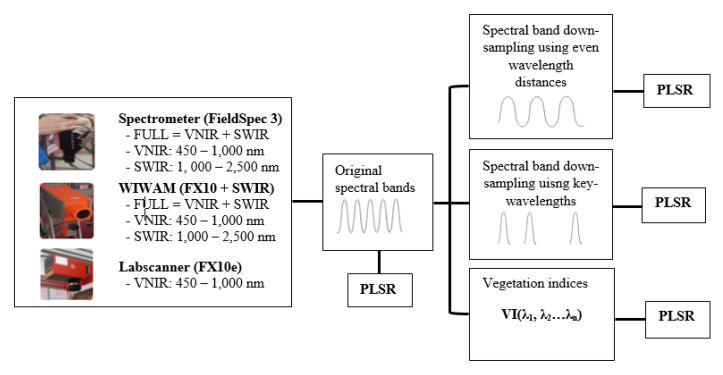
Data transformation and PLSR. First, the pre-processed data from different sensors was separated into the different ranges of visible and near-infrared (VNIR), short-wave infrared wavelengths (SWIR) and VNIR + SWIR (FULL) for PLSR. Then in each range, the data was re-sampled to different spectral bands or transformed into different VIs for PLSR.

To answer the research questions in Section 1, the pre-processed reflectance data were transformed into different wavelength ranges and spectral bands for modelling (Figure 2). First, the reflectance data from the FieldSpec 3 spectrometer, phenotyping platform (FX10 + SWIR) and labscanner (FX10) were separated into three different wavelength ranges of FULL (VNIR+SWIR), VNIR and SWIR to train and validate the PLSR models. Second, to test the effects of spectral resolution, the reflectance data of the different sensors and wavelength ranges was spectrally re-sampled to the evenly distributed spectral bands of 10 nm to 100 nm FWHM for training and validation. The spectral band re-sampling algorithm treats a sensor as having Gaussian spectral response functions for each of its spectral bands. A source sensor band (the original band) will contribute to any destination band (transformed band) where there is overlap between the FWHM of the response functions of the two bands [24]. Third, using the PLSR coefficient of the model trained by the FULL range spectrometer data, the key-wavelengths were found through the magnitudes of PLSR coefficients which represent the contributions of the different wavelengths to the model [8]. The PLSR coefficients of the five-fold cross-validation were averaged and the wavelengths corresponding to the local maximum or minimum of PLSR coefficient were considered to be key-wavelengths which had the highest correlation to the N% values. The selected key-wavelengths were used as the centres of the destination spectral bands for spectral band re-sampling, and the resulting key-bands were used for PLSR. To evaluate the models’ sensitivity to bandwidth, both narrow bandwidths (NBs) and broad bandwidths (BBs) were tested. The narrow bandwidths were equal to the original bandwidths output from the sensors whose FWHM were 1 nm, 5.5 nm, 12 nm and 5.5 nm for the ASD FieldSpec 3 spectrometer, FX10 and SWIR of the phenotyping platform and FX10 of the labscanner respectively. The FWHM of the broad bands was set to reach half of the distance to their neighbouring key-wavelengths.

Finally, for comparison, some well-known vegetation indices (VIs) previously used to measure the N content of plants were tested using the spectrometer data. Tan, et al. [10] compared 18 VIs using hyperspectral data and found that the normalised red edge area index (NREAI) proposed by Gong, et al. [25] had the best correlation with the leaf N accumulation (LNA) of wheat. NREAI has the form of (*SD*_r_ − *SD*_b_)/(*SD*_r_ + *SD*_b_), where *SD*_b_ is the integration of the first-order derivative values in the blue-edge covering 490 nm to 530 nm and *SD*_r_ is the integration of the those in the red-edge from 670 nm to 737 nm. The least square regression was conducted in the NREAI values. As one of the most widely used indices for estimating crop vigour, the normalised difference vegetation index (NDVI) was also investigated [26,27]. NDVI is a function of the intensity or reflectance of two wavelengths *λ*_i_ and *λ*_j_ and for hyperspectral data, many different forms of NDVI have been reported width different wavelengths and bandwidths. To find the best NDVI form, an NDVI matrix was calculated for each spectral signature of the spectrometer, which includes all possible combinations of *λ*_i_ and *λ*_j_ in the range of 400 nm to 2400 nm. Least square regression was conducted using each of the NDVI (*λ*_i_, *λ*_j_).

Five metrics were used to evaluate the accuracy of the PLSR models in different aspects, including the coefficient of determination (*R*^2^), root mean square error (*RMSE*), bias (*Bias*), mean absolute error (*MeanABS*) and median absolute error (*MediaABS*). The definitions of the metrics are shown in Equations (2)–(6), where *n* is the number of samples; *y_i_* is the *i*th reference value of N%; ${\hat{y}}_{i}$ is the *i*th estimated value using a trained model.

(2) $$R^{2}=1-\frac{\sum_{i=1}^{i=n}{\left(y_{i}-{\hat{y}}_{i}\right)}^{2}}{\sum_{i=1}^{i=n}{\left(y_{i}-{\bar{y}}_{i}\right)}^{2}}$$

(3) $$RMSE=\sqrt{\frac{1}{n}\sum_{i=1}^{i=n}{\left(y_{i}-\hat{y}\right)}_{i}{}^{2}}$$

(4) $$Bias=\frac{1}{n}\sum_{i=1}^{i=n}\left({\hat{y}}_{i}-y_{i}\right)$$

(5) $$MeanABS=\frac{1}{n}\sum_{i=1}^{i=n}\left|{\hat{y}}_{i}-y_{i}\right|$$

(6) $$MedianABS=\mathrm{median}\;\mathrm{of}\;\left|{\hat{y}}_{i}-y_{i}\right|$$

## 3. Results and Discussion

### 3.1. A Hyperspectral Camera with a Proper System Setup Can Provide Comparable Accuracy in N Measurement to a Non-Imaging Spectrometer with Contact Measurement

**Figure 3:**
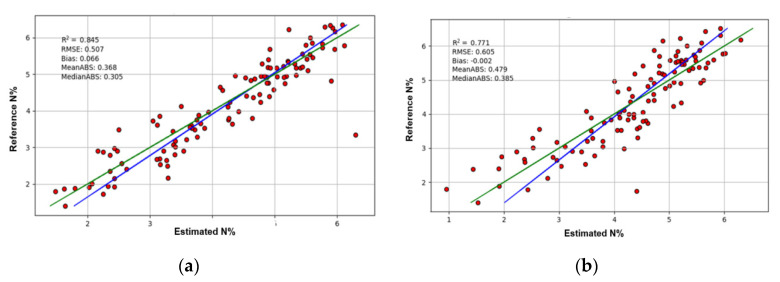
The first-fold validation results of the PLSR models. The red dots are the results of validation. The green line is the ideal regression line and the blue line is the real regression line. (**a**) The first-fold validation result of the model of the original FULL range data of the ASD FieldSpec 3 spectrometer; (**b**) The first-fold validation result of the model of the original FULL range data of the FX10 and SWIR cameras in the phenotyping platform.

**Figure A2:**
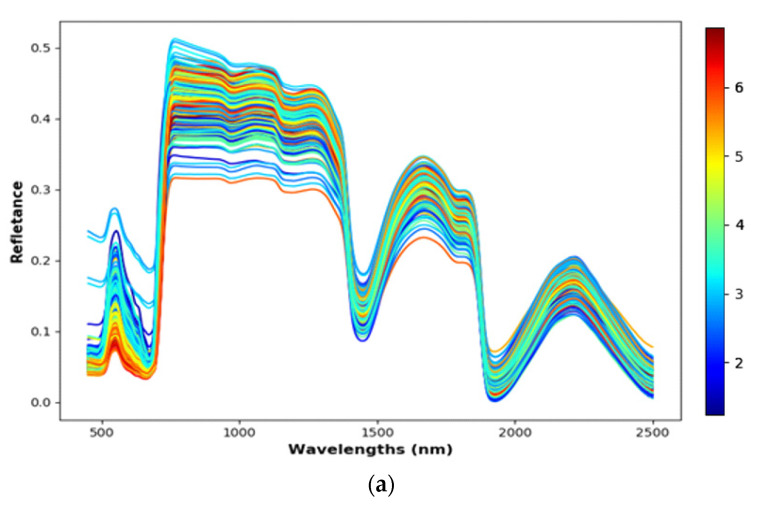

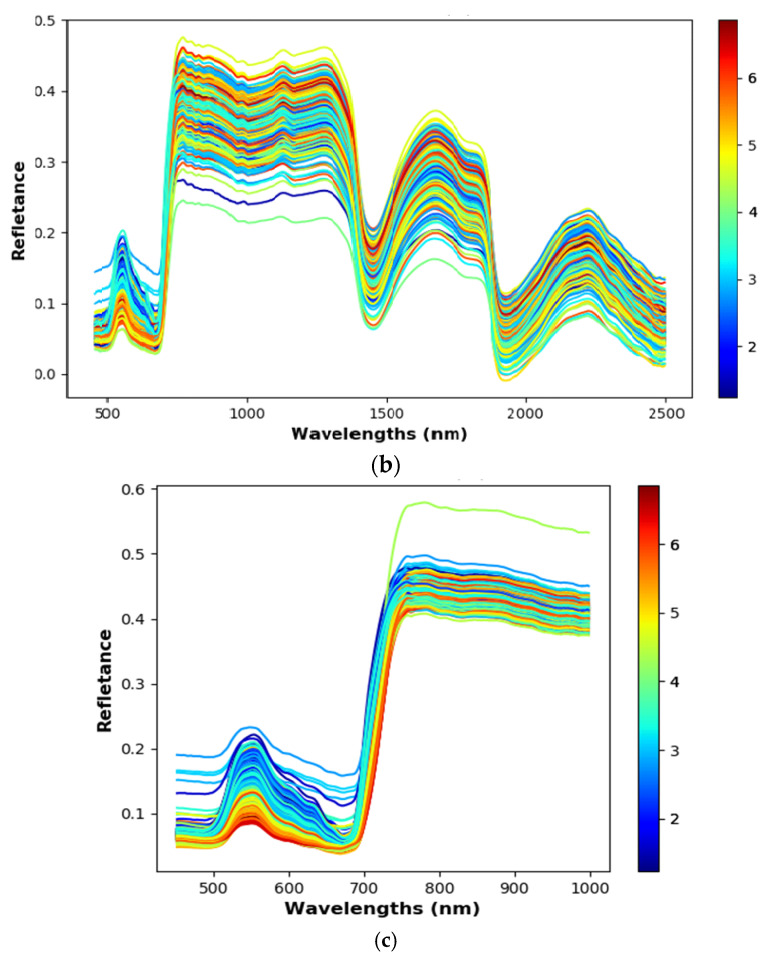
The reflectance data. The different colours represent different levels of N%. (**a**) The reflectance data of ASD FieldSpec 3. (**b**) The reflectance data of FX10 and SWIR cameras in the phenotyping platform. (**c**) The reflectance data of the FX10 labscanner.

**Figure A3:**
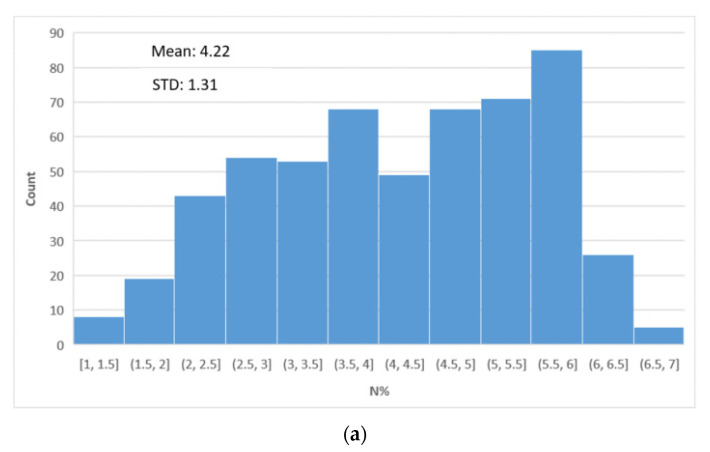

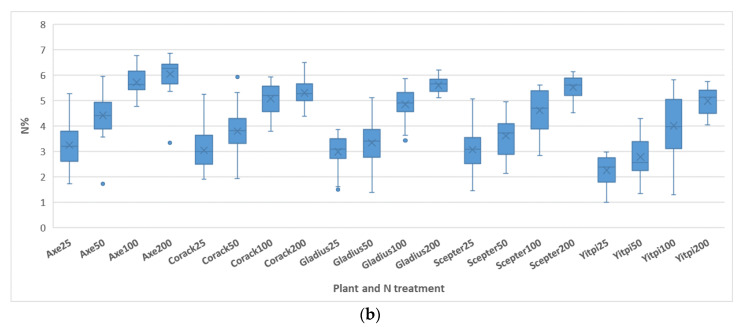
Distributions of reference N% values (**a**) Distribution of all N% values. The mean N value is 4.22% with a standard deviation of 1.31%. The distribution has the highest peak in the range of 5.5% to 6.0%. (**b**) Box & whisker plot of the N% values according to the plants and N treatments.

**Figure A4:**
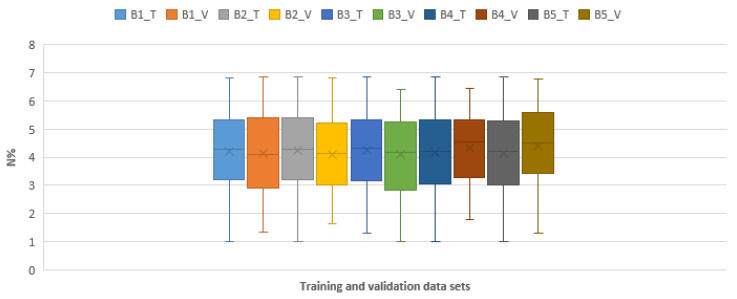
Distributions of reference N% values for the five-fold cross-validation. B1_T represents the training data of batch 1 and B1_V represents the validation data of batch 1, so on and so forth. This figure shows that the distributions of the training and validation data set are close to normal distributions and there are no clear statistical differences in the training and validation dataset; thus the data is reasonable for training and validating the models.

The reflectance data of the hyperspectral sensors are plotted in Figure A2 and the distributions of the reference N% values are shown in Figure A3. Table 1 lists the average *R*^2^ values of the five-fold cross-validation of the models trained using the data of different sensors with or without data transformation. For conciseness, only the first-fold validation results of the models of the original data in the FULL wavelength ranges are plotted in Figure 3.

The *R*^2^ value of the model of the FULL-range ASD FieldSpec 3 data without transformation was 0.86, which was better than 0.77 of the FX10 and SWIR cameras (Table 1). Similarly, after different data transformations, the models of the ASD FieldSpec 3 outperformed that of the FX10 and SWIR cameras of the phenotyping platform, regardless of the FULL, VNIR or SWIR ranges. There were a few exceptions to this rule after spectral re-sampling, however, this does not change the overall trend that the contact measurement using the spectrometer can provide more reliable data for PLSR than the phenotyping system. In the phenotyping system, the FX10 and SWIR cameras were set up to image whole plants. The wheat plants have thin, long and curved leaves with complex geometrical properties. Thus, many factors could affect the hyperspectral images, such as shadow, working distance, the surface angles of leaves and the transmittance and reflection from neighbouring leaves [15,16,28,29,30]. Part of these effects was reduced by binding the target leaves to the leaf-beds, however, this could not guarantee a perfect correction. When performing close-range hyperspectral imaging of whole plants, an ideal solution to reduce the negative factors from the complex geometrical features of plants is using diffused illumination, however, this is not practical for most plant phenotyping systems because of system cost and complexity. The most feasible solution would be using data pre-processing algorithms to mitigate the negative effects [21,22,23,31,32], and this is considered productive future work.

However, when considering the FX10 labscanner, it shows a different trend. The models using the VNIR imaging data of the labscanner outperformed that of the VNIR camera of the phenotyping platform and were as good as or better than that of the FieldSpec 3 spectrometer. Using the data without transformation, the *R*^2^ values are 0.76, 0.75 and 0.71 for the FX10 labscanner, FiedSpec 3 and FX10 of the phenotyping system respectively. It displayed the same trend after data transformation. This result shows that the performances of imaging systems are heavily affected by system setups. When imaging with the labscanner, the leaves with the leaf-beds were laid on the conveying tray to minimise the effects of working distance, surface angle, transmittance and reflection. Further, the reflectance data of the labscanner was averaged for each leaf, providing a measurement more indicative of the entire leaf in comparison to the point-measurement of the spectrometer. This result indicates that with proper system setups to reduce the negative factors from plant geometry, hyperspectral imaging systems can perform as well as or better than non-imaging spectrometers with contact measurement.

**Figure A5:**
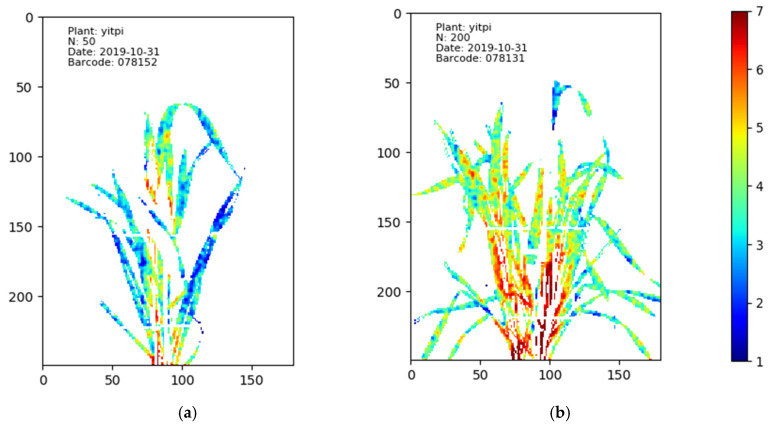
The N distribution maps of Yitpi on 31 October 2019 with the different N treatments. The best performing model trained using the SWIR camera data was used to create N distribution maps. The different colours represent different levels of N%. In image (**a**), the plant treated with 50 mg/kg N has more blue pixels than the plant treated with 200 mg/kg N shown in image (**b**), saying that the leaves shown in (**a**) have lower N content than that shown in (**b**).

**Figure A6:**
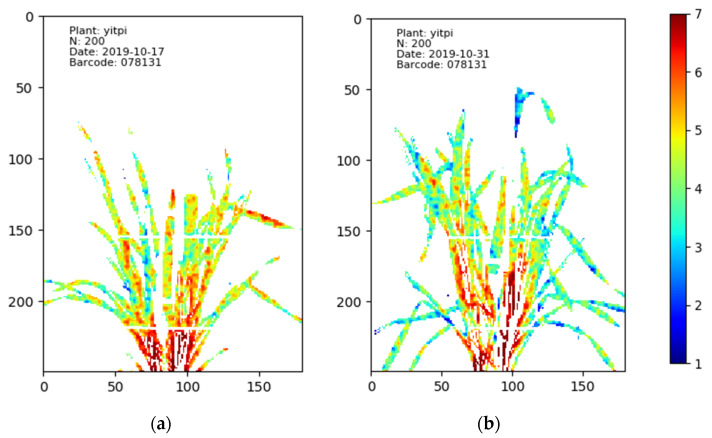
The N distribution maps of Yitpi on different days with the same N treatments. Image (**a**) shows the plant on 17 October when it was still young. In the different parts, most of the pixels show orange or green colour, indicating sufficient N was roughly evenly distributed in the leaves. In contrast, image (**b**) shows the same plant at an older stage on 1 December. Because the N was insufficient to supply all of the leaves, some old leave and the tips of young leaves lacked enough N, changing to blue.

In previous studies, researchers using similar sensors and modelling methods as those in this study have achieved different accuracies when measuring N in wheat. Silva-Perez, et al. [9] achieved *R*^2^ = 0.93 for N per unit leaf area using a FieldSpec 3 spectrometer and Bruning, et al. [4] obtained *R*^2^ = 0.66 for N% using a combination of an FX10 and SWIR camera. These results indicate that our sensors and modelling methods can provide sufficient or satisfactory accuracy for N measurement. To the authors’ knowledge, this is the first study to compare the performances of a spectrometer and hyperspectral cameras for measuring N content in wheat leaves at close range. Though the spectrometer integrated the VNIR and SWIR sensors and it is convenient in both laboratory and field conditions, the hyperspectral cameras have many advantages over the non-imaging sensor. If automation is used in the system setup, the data collection using hyperspectral cameras requires less human involvement, and therefore they are more suitable to high-throughput plant phenotyping. The hyperspectral cameras can provide two-dimensional images of plants, which is valuable for combined spectral and morphological analysis. The N maps created using the trained models can be used to visualise the N distribution within the plants (refer to Figure A5 and Figure A6 and the work of Bruning, et al. [4]). However, in proximal sensing, hyperspectral images are sensitive to many uncontrolled factors, such as leaf angles and shadow. Furthermore, to collect FULL range hyperspectral images, multiple sensors have to be used, increasing system complexity and cost. In the phenotyping platform, the FX10 and SWIR cameras have their individual optical systems, operating at different spatial and spectral resolutions. Though sensor fusion is possible in some cases [23,33], it is computationally expensive and not reliable in all conditions.

### 3.2. InGaAs or MCT Photonic Detectors are More Sensitive to N Content in Wheat Leaves than CCD or CMOS Detectors

Most of the commercially available hyperspectral sensors use either CCD/CMOS photonic detectors working in the VNIR range or InGaAs/MCT detectors operating in the SWIR range. It is therefore important to determine which wavelength range is required when selecting sensors for each application. However, limited studies have been reported which compare the performances of different wavelength ranges for measuring N content in wheat. Bruning, et al. [4] reported that, compared with using VNIR data only, using the FULL wavelength range from 400 nm to 2500 nm for PLSR can marginally improve the accuracy for N measurement in wheat.

In this study, the *R*^2^ values for the models using the spectrometer data without transformation were 0.86, 0.84 and 0.75 in the FULL, SWIR and VNIR range respectively (Table 1). The models of the FULL and SWIR ranges had similar performances and achieved higher *R*^2^ values than those of the VNIR data. Similar trends were observed after data transformation but with a few exceptions. The performances of the cameras of the phenotyping platform showed the same pattern as the spectrometer. This result indicates that hyperspectral sensors with InGaAs or MCT detectors working in the SWIR wavelength range are more suitable for measuring N content in wheat leaves than VNIR cameras using CCD or CMOS detectors. However, since reflectance in the SWIR region is highly affected by both water and protein content [28,34,35], the effects of varying water content should be further investigated. One possible explanation of the better performance of the SWIR wavelength range over the VNIR range is because there are more key-wavelengths located in the SWIR range which are highly correlated to N content in plants (see Section 3.4).

### 3.3. N Content can be Measured at Reduced Spectral Resolutions

When spectrally down-sampling the FULL data of the ASD FieldSpec 3 spectrometer from FWHM 10 nm to 80 nm at a 10 nm interval, the models performed similarly to those models without data transformation (Table 1). The SWIR models returned similar results regardless of the down-sampling. However, the VNIR models were sensitive to the spectral resolution at certain levels after FWHM was wider than 30 nm. The camera model had a similar trend: the models using the FULL and SWIR data were not affected by spectral re-sampling when the bandwidth was narrower than 50 nm while the models of VNIR data started to lose accuracy after the FWHM reached 30 nm. This suggests that the PLSR models are not very sensitive to the spectral resolution of the reflectance data for quantifying N% in wheat leaves. This study showed that low-cost hyperspectral sensors with coarse spectral resolution could be used to measure N content of wheat without a significant drop of accuracy.

### 3.4. Wavelengths most Correlated to N Content in Wheat

**Figure 4:**
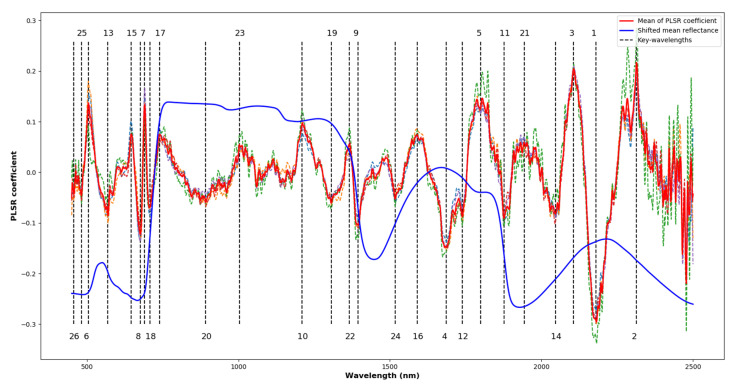
The PLSR coefficients and the key-wavelengths identified. The dotted curves in random colours are the values of the PLSR coefficients in the different folds of validation. The red curve is the average values of the PLSR coefficients in cross-validation and the dotted lines with numbers mark the locations and order of contribution of the key-wavelengths. The vertically shifted average reflectance is plotted in blue.

High-dimensional hyperspectral data are highly correlated, resulting in unnecessarily large data size and overfitting of regression models [36]. Thus, it is essential to determine the key-wavelengths to reduce the redundancy of data and improve model accuracy. Figure 4 plots the PLSR coefficient versus wavelength and Table 2 lists the key-wavelengths. There were 26 key-wavelengths identified between 400 nm and 2400 nm. The SWIR spectrum had a greater contribution than the VNIR spectrum for measuring N content. Out of the 26 key-wavelengths, 16 were in the SWIR range. In the top ten, seven were in the SWIR range and the top five were all in the SWIR range.

These key-wavelengths were used as the centres for spectral band re-sampling and the resulting data were used for PLSR to evaluate whether reduced datasets using only the key-wavelengths result in a loss of performance. In the FULL range data of the ASD FieldSpec 3 spectrometer, using only the top 26 or 20 key-wavelengths, the performances of these models did not drop, regardless of NB or BB (Table 1). From the top 16 to top five key-wavelengths, the performances dropped gradually when reducing the number of wavelengths or increasing the bandwidths. In the SWIR or VNIR range, the models using the key-wavelengths had poorer accuracy than the FULL models, however, using the top 16 in SWIR or top 10 in VNIR, the performances of the models were not significantly affected. In the imaging data, the responses of the models to the key-wavelength spaces were similar to those of the spectrometer data.

Previous studies have reported 19 of the key-wavelengths identified here as being related to physiological, biochemical or nutritional traits (Table 2). Since the key-wavelengths could shift slightly depending on different plants and environments, reported bands matching the key-wavelengths identified within a 25 nm tolerance were considered. The most significant key-wavelength with the highest regression coefficient is located at 2180 nm corresponding to the centre of the protein band as reported by [35]. The identified wavelengths at 2313 nm, 2106 nm, 1686 nm and 1800 nm are close to 2300 nm, 2130 nm, 1690 nm and 1770 nm respectively, which have been reported to relate to N-H stretch, protein and N content [37]. A significant band centred at 505 nm in the visible spectrum is mainly influenced by leaf pigments of chlorophyll or carotenoids [38]. The bands centred at 690 nm and 676 nm are located at the red-edge and affected by electron transition and chlorophyll [35,37]. The key-wavelengths between 1210 nm to 1395 nm, 1590 nm, 1739 nm and 1800 nm were not found to correspond to previously identified chemical bonds or traits and require further investigation.

### 3.5. Cross-Sensor Validation

**Figure 5:**
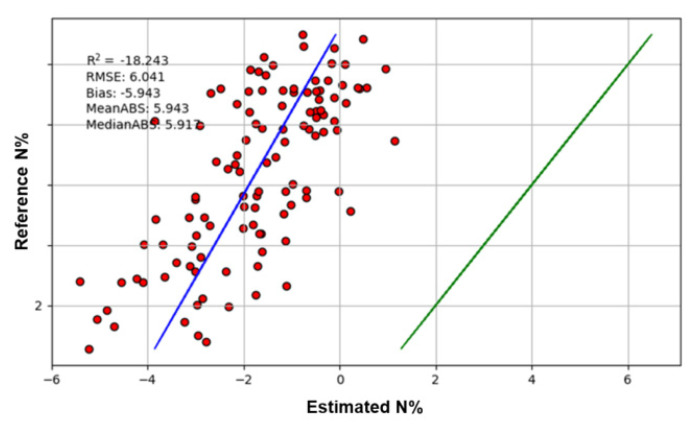
The result of cross-sensor validation. The red dot shows the results of validation. The blue line is the true regression line and the green line is the ideal regression line.

As the application of hyperspectral imaging increases, it would be beneficial to apply previously developed models using different sensors without the need to repeat extensive modelling work. To the authors’ knowledge, there are few reports where previously trained models of different sensors have been re-used. Here, we applied the model developed by the spectrometer data unaffected by leaf geometry to hyperspectral images. Unfortunately, the spectrometer model did not show promise for cross-sensor application. The validation has a large bias of −5.94 and *R*^2^ value of −18.24 (Figure 5). However, the estimated N% values have a linear relationship with the reference values, indicating that there is potential to re-calibrate the model to improve accuracy. Cross-sensor modelling would be of future interest to allow the re-use of previously established models.

### 3.6. Vegetation Indices

**Figure 6:**
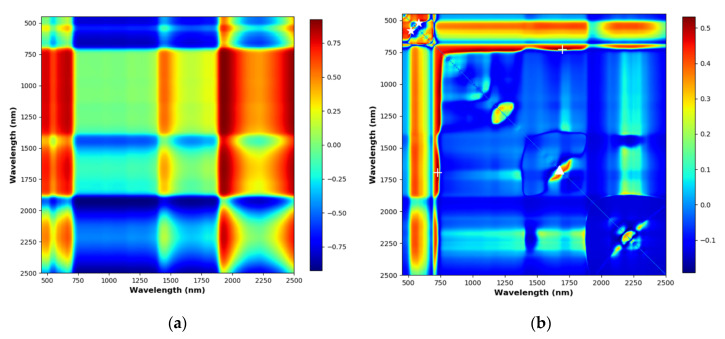
NDVI and *R*^2^ matrices. The different colour indicating the different values of NDVI or *R*^2^. (**a**) An NDVI matrix showing all possible wavelength combinations of a reflectance data from the ASD FieldSepc 3. (**b**) The *R*^2^ matrix of NDVI for validating the PLSR models.

Only narrow-band NDVI was tested for estimating N% since narrow-band NDVI has been shown to perform significantly better than broad-band NDVI, which can suffer from serious problems of saturation [37]. Different band combinations can be used to calculate NDVI depending on sensors, vegetation and soil types [42]. In this study, all possible narrow-band NDVI of the spectrometer data in the wavelength range of 400 nm to 2500 nm with 1 nm FWHM were tested. Figure 6a shows an example NDVI matrix showing the NDVI values for all possible combinations of wavelengths of a spectral signature in the range of 400 nm to 2500 nm. The *R*^2^ values of validation were calculated to form a *R*^2^ matrix of NDVI (Figure 6b). The best-performing NDVI found across the FULL, SWIR and VNIR wavelength ranges were NDVI_full_ (1696 nm, 729 nm), NDVI_swir_ (1672 nm, 1647 nm) and NDVI_vnir_ (519 nm, 582 nm) respectively. The corresponding *R*^2^ values were 0.53, 0.44 and 0.53, which did not show strong correlations to N%. The NREAI index was calculated and the corresponding *R*^2^ for validation was 0.55, which performed similarly to the best NDVI index. None of the VIs tested performed as well as the hyperspectral models established in this study, highlighting the gain in estimation accuracy with a sufficient number of spectral bands.

## 4. Conclusions

This study investigated the performance of different hyperspectral sensors for measuring N content in wheat leaves. We answered four important research questions relevant to the field of plant phenotyping. First, the ASD FieldSpec 3 spectrometer with a leaf-clip can be used to measure N content in wheat leaves with high accuracy. However, the point-measurement cannot represent the spectral signature of a whole leaf or plant. When sensing flat leaves at close range, a hyperspectral camera with sufficient spatial resolution can outperform a non-imaging spectrometer working in the same wavelength range with contact measurement. Leaf surface angle, working distance, shadow and the reflectance and transmittance of neighbouring leaves can impact the accuracy of measurement when imaging a whole plant. Diffused illumination or data pre-processing methods should be considered to reduce these negative factors. Although using hyperspectral images to conduct proximal sensing of N in plants is challenging, our study demonstrated that hyperspectral imaging technology is a promising approach for non-destructive and high-throughput measurement of N in plants. Second, for N measurement in wheat, hyperspectral sensors with photonic detectors of InGaAs or MCT working in the SWIR wavelength range of 1000 nm to 2500 nm outperformed the cameras with CCD or CMOS detectors sensitive to the VNIR wavelengths from 400 nm to 1000 nm. However, the effects of plant water content on N estimation in the range of 1000 nm to 2500 nm should be further investigated. Users need to consider the types of photonic detectors when selecting hyperspectral sensors for their applications. Third, the PLSR models were not very sensitive to the spectral resolution. Depending on different sensors and wavelength ranges, the hyperspectral data can be spectrally down-sampled to a certain extent without losing accuracy. Down-sampled data can significantly reduce the data size and increase computational speed. Using low-cost hyperspectral sensors with coarser spectral resolutions to measure N content in plants is plausible. Finally, a total of 26 key-wavelengths between 400 nm and 2400 nm were found to be highly influential for measuring N in wheat. Of these, 16 key-wavelengths were in the SWIR range and ten in the VNIR range. The protein band centred at 2180 nm played the most significant role. Understanding the key-wavelengths is significant to reduce data size, speed up computation and aid in designing low-cost and compact multispectral sensors for field applications. The conclusions of this study provide guidance for proximal sensing of N in plants using hyperspectral sensors, including sensor selection and model development. In future work, more wheat varieties, soil and water treatments should be included to ensure model robustness and transferability. This study was limited to the controlled environment of a plant phenotyping platform and further work should compare controlled environment and field results.

## Figures and Tables

**Table 1:** The average *R*^2^ for validating the PLSR models. The rows include three sections. The first section demonstrates the results of the models built from the original data and the spectrally re-sampled data with even wavelength distances and full width at half maximum (FWHM). The second section lists the results of the models using only the key-wavelengths, including both narrow bandwidth (NB) and broad bandwidth (BB). The third part shows the regression results of some well-known VIs. The bold fonts highlighted the top three *R*^2^ values in corresponding sensors and wavelength ranges. N/A stands for “not available”.

Different Spectral Bands and VI		ASD FieldSpec 3			FX10 + SWIR			FX10
		FULL	SWIR	VNIR	FULL	SWIR	VNIR	VNIR
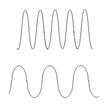 Spectral band re-sampling with evenly distributed wavelength centres and bandwidths	No transformation	**0.86**	**0.84**	**0.75**	**0.77**	**0.76**	**0.71**	**0.76**
	FWHM = 10 nm	**0.84**	**0.85**	**0.74**	**0.78**	**0.76**	**0.71**	**0.76**
	FWHM = 20 nm	**0.86**	**0.86**	**0.74**	**0.78**	**0.74**	**0.71**	**0.77**
	FWHM = 30 nm	**0.85**	**0.86**	**0.73**	**0.77**	**0.76**	**0.68**	**0.73**
	FWHM = 40 nm	**0.85**	**0.84**	0.69	**0.77**	**0.74**	**0.68**	0.69
	FWHM = 50 nm	**0.86**	**0.84**	0.66	**0.76**	**0.74**	0.66	0.70
	FWHM = 60 nm	**0.85**	**0.84**	0.66	0.75	0.69	0.65	0.68
	FWHM = 70 nm	**0.84**	**0.84**	0.60	0.73	0.72	0.64	0.67
	FWHM = 80 nm	**0.84**	**0.84**	0.61	0.72	0.71	0.64	0.66
	FWHM = 90 nm	0.80	0.76	0.61	0.73	0.68	0.64	0.65
	FWHM = 100 nm	0.74	0.82	0.62	0.67	0.70	0.62	0.66
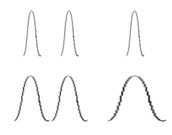 Spectral band re-sampling using the key-wavelengths with narrow or broad bandwidths	Top 26 NB	**0.86**	N/A	N/A	**0.77**	N/A	N/A	N/A
	Top 26 BB	**0.84**	N/A	N/A	**0.76**	N/A	N/A	N/A
	Top 20 NB	**0.85**	N/A	N/A	**0.77**	N/A	N/A	N/A
	Top 20 BB	**0.84**	N/A	N/A	0.75	N/A	N/A	N/A
	Top 16 NB	**0.85**	0.83	N/A	**0.76**	**0.76**	N/A	N/A
	Top 16 BB	0.81	0.82	N/A	0.74	**0.74**	N/A	N/A
	Top 10 NB	0.78	0.83	0.70	0.66	0.68	**0.69**	0.72
	Top 10 BB	0.64	0.78	0.69	0.62	0.66	**0.69**	0.72
	Top 5 NB	0.64	0.64	0.69	0.60	0.61	0.65	0.72
	Top 5 BB	0.44	0.43	0.67	0.48	0.48	0.57	0.71
VI (λ_1_, λ_2_…λ_n_)	NREAI	N/A	N/A	0.55	N/A	N/A	N/A	N/A
	NDVI (1696, 729)	0.53	N/A	N/A	N/A	N/A	N/A	N/A
	NDVI (519, 582)	N/A	N/A	0.53	N/A	N/A	N/A	N/A
	NDVI (1672, 1647)	N/A	0.44	N/A	N/A	N/A	N/A	N/A

**Table 2:** Physiological, biochemical and nutritional traits reported previously related to the key-wavelengths identified in this study.

Order	PLSR Coefficient	Key-Wavelength (nm)	Physiological, Biochemical and Nutritional Traits Relate to the Key-Wavelengths (± 25 nm)
26	0.043	456	460 nm relates to chlorophyll a,b and electron transition [35].
25	0.045	482	495 nm is the longer wavelength portion of the blue band; crop-to-soil reflectance ratio is minima for blue and green bands [37].
6	0.137	505	520 nm is mainly influenced by leaf pigments, such as chlorophyll or carotenoids [38].
13	0.085	569	540 nm to 550 nm is the peak of the green band and the maximum first-order derivative in the visible spectrum [37].
15	0.075	646	660 nm relates to N [39].
8	0.122	676	670 nm relates to electron transition and chlorophyll a,b [35].
7	0.133	690	668 nm to 696 nm is chlorophyll absorption maxima [37].
18	0.070	708	In the red-edge range in which the slope and position are salient features to distinguish vigorous plants from others [37,40].
16 (17)	0.074	740	
20	0.059	892	845 nm is NIR shoulder; 920 nm is the peak of NIR [41]; 910 nm relates to C-H stretch and protein [35].
23	0.054	1003	1020 nm relates to N-H stretch and protein [35].
10	0.096	1210	Not previously reported.
19	0.061	1307	Not previously reported.
22	0.055	1366	Not previously reported.
9	0.106	1395	Not previously reported.
24	0.054	1517	1510 nm relates to N-H stretch, protein and N [35,39].
17 (16)	0.074	1590	Not previously reported.
4	0.148	1686	1690 nm relates to C-H stretch, protein and N [35].
12	0.087	1739	Not previously reported.
5	0.145	1800	Not previously reported.
11	0.092	1876	1900 nm relates to O-H stretch [35].
21	0.059	1943	1940 nm relates to N-H deformation, protein and N [35].
14	0.083	2046	2060 nm relates to N-H bend, protein and N [35].
3	0.205	2106	2130 nm relates to N-H stretch and protein [35].
1	0.297	2180	2180 nm relates to N-H bend, protein and N [35].
2	0.217	2313	2300 nm relates to N-H stretch, protein and N [35].

## Supplementary Materials

The pre-processed data and the Python codes for the demonstration are available at https://adelaide.figshare.com/articles/public_data_for_wheat_n_experiment/12502160.

## Appendix A

**Table A1:** Wheat varieties used in this study.

Wheat Plants	Description
Gladius	Waxy leaves, performs well under drought stress
Axe	No-waxy gladius, very early maturing, performing best when suffering from drought stress
Scepter	Greenest of Wyalkatchem, one of the most widely-grown wheat varieties in southern Australia
Corack	Pale coloured Wyalkatchem, very high and stable grain yield
Yitpi	Longer maturity, Western Australian industry standard for early sowing

**Table A2:** Data errors and outliers.

Items	No.
Laboratory analysis error of N %	42
Outliers of N%	8
Leaves fell out of the leaf-beds	3
Green plant segmentation error	2
Hyperspectral image error	2
**Total valid samples**	**543**
**Total samples**	**600**
